# Relationships among ergot alkaloids, cytochrome P450 activity, and beef steer growth

**DOI:** 10.3389/fchem.2015.00016

**Published:** 2015-03-11

**Authors:** Charles F. Rosenkrans, Nicholas S. Ezell

**Affiliations:** Department of Animal Science, University of ArkansasFayetteville, AR, USA

**Keywords:** fescue toxicosis, predictive biology, cattle

## Abstract

Determining a grazing animal's susceptibility to ergot alkaloids has been a research topic for decades. Our objective was to determine if the Promega™ P450-Glo assay could be used to indirectly detect ergot alkaloids or their metabolites in urine of steers. The first experiment validated the effects of ergot alkaloids [0, 20, and 40 μM of ergotamine (ET), dihydroergotamine (DHET), and ergonovine (EN)] on human CYP3A4 using the P450-Glo assay (Promega™ V9800). With this assay, luminescence is directly proportional to CYP450 activity. Relative inhibition of *in vitro* cytochrome P450 activity was affected (*P* < 0.001) by an interaction between alkaloids and concentration. That interaction resulted in no concentration effect of EN, but within ET and DHET 20 and 40 μM concentrations inhibited CYP450 activity when compared with controls. In experiment 2, urine was collected from Angus-sired crossbred steers (*n* = 39; 216 ± 2.6 days of age; 203 ± 1.7 kg) after grazing tall fescue pastures for 105 days. Non-diluted urine was added to the Promega™ P450-Glo assay, and observed inhibition (3.7 % ± 2.7 of control). Urine content of total ergot alkaloids (331.1 ng/mg of creatinine ± 325.7) was determined using enzyme linked immunosorbent assay. Urine inhibition of CYP450 activity and total alkaloids were correlated (*r* = −0.31; *P* < 0.05). Steers were genotyped at CYP450 single nucleotide polymorphism, C994G. Steer genotype affected (*P* < 0.03) inhibition of CYP450 activity by urine; heterozygous steers had the least amount of CYP450 inhibition suggesting that genotyping cattle may be a method of identifying animals that are susceptible to ergot alkaloids. Although, additional research is needed, we demonstrate that the Promega™ P450-Glo assay is sensitive to ergot alkaloids and urine from steers grazing tall fescue. With some refinement the P450-Glo assay has potential as a tool for screening cattle for their exposure to fescue toxins.

## Introduction

Tall fescue [*Lolium arundinaceum* (Schreb.) S. J. Darbyshire] infected with the endophytic fungus (*Neotyphodium coenophialum*) is responsible for the production of ergot alkaloids which have a significant economic impact on beef cattle. Consumption of those mycotoxins is known to lower average daily gain, negatively impact reproductive traits, decrease parasite resistance, and reduce heat tolerance, all of which drive up the cost of production (for review see Strickland et al., 2011). Ergot alkaloids have an interesting relationship with animal cytochrome P450 (CYP) enzymes; CYP have been shown to metabolize ergot alkaloids via hydroxylation, but CYP enzyme activity is inhibited by ergot alkaloids (Althaus et al., 2000; Settivari et al., 2006). Furthermore, CYP is a key enzyme in the biosynthetic pathway for ergot alkaloids (Haarmann et al., 2006).

Current methods for detecting ergot alkaloid concentrations in bodily fluids are limited. Initially, chromatographic methods had relatively high detection limits (Yates and Powell, 1988; Rottinghaus et al., 1991; Moubarak et al., 1996); however, more recent advances in chromatographic and detection methods have vastly improved the sensitivities of the assays (Lehner et al., 2005; Smith et al., 2009; Foote et al., 2012; Wang et al., 2013). Enzyme linked immunosorbent assay is a faster method for detection of ergot alkaloids and has been used to screen livestock exposure to fescue toxins (Shelby and Kelley, 1990; Hill and Agee, 1994). Recently, we demonstrated that the Promega™ P450-Glo assay could be used to assess ergot alkaloid content of tall fescue extracts (Moubarak et al., 2012). The Glo-assay is relatively easy with a quick protocol which makes it a convenient tool for biologically-relevant indirect alkaloid detection. Our objectives were to validate our previous findings related to ergot alkaloid inhibition of CYP450 activity, and determine if urine collected from steers grazing tall fescue inhibited CYP450 activity *in vitro*.

## Materials and methods

### Experiment 1

#### CYP450 analysis

Ergotamine (ET), dihydroergotamine (DHET), and ergonovine (EN) were dissolved in methanol (100%) resulting in final assay concentrations of 0, 20, and 40 μM. Enzyme activity for CYP450 was assayed using the Promega™ P450-Glo assay (product # V9920; Cali et al., 2009). Briefly, sample (12.5 μL) was added in triplicate to a 96-well plate, followed by CYP3A4 solution (12.5 μL), and incubated at 37°C for 10 min. Reaction was initiated by addition of NADPH regeneration system (25 μL) and plate was incubated 30 min at 37°C. Luciferin Detection Reagent (50 μL) was added and plate was incubated at 20°C for 20 min. Luminescence was recorded with a luminometer (Perkin-Elmer, Victor 1420 multilabel counter) using no filters and an integration time of 1 s/well.

#### Statistical analysis

Luminescence data for each alkaloid were analyzed independently using mixed procedures. Main effects were replicate, alkaloid, and concentration; response variable was CYP450 activity as indicated by luminescence. If *F*-tests were significant (*P* < 0.05), multiple *t*-tests were used to separate means. Results are presented as a percent of CYP450 activity with zero alkaloid present.

### Experiment 2

#### Steer grazing

The University of Arkansas Animal Care and Use Committee approved all animal procedures (protocol # 04024). Healthy, fall-born Angus-sired crossbreed steers (*n* = 39; 203 ± 27 kg; 216 ± 2.6 days of age) were weaned, vaccinated, and treated with anthelmintic in May. Fourteen days after weaning, steers were randomly assigned to a pasture of either Kentucky 31 (K31; four pastures; *n* = 20 steers) or HiMag4 (HM4; four pastures; *n* = 19 steers) for a summer grazing trial. Pastures and tall fescue cultivars were as previously described (Nihsen et al., 2004). During the post-weaning grazing trial (105 days) steers received a daily supplement (1.8 kg/day; 80% ground corn: 20% soybean meal; 12% CP). During the grazing period steers had free access to water and minerals. Steers remained healthy throughout the trial and were not treated with pharmaceuticals.

#### Sample collection

At weaning, approximately 10 mL of blood was collected from each steer, stored at 5°C until centrifuged at 1200 × *g* for 30 min, serum was decanted and stored at −20°C until hormone [cortisol, prolactin, and insulin-like growth factor 1 (IGF-1)] concentrations were determined by validated radioimmunoassay. At day 105, each animal was weighed and a urine sample collected. Urine samples were stored at −20°C until assayed. Total alkaloids were determined by ELISA (Agrinostics Ltd. Co., Watkinsville, GA). Inhibition of CYP450 was determined using the assay (Promega™ P450-Glo) described above using 12.5 μL of non-diluted urine in each reaction test.

#### Cytochrome P4503A28 genotyping

Steers were genotyped at CYP3A28 single nucleotide polymorphism (SNP), C994G, using methods previously described (Sales et al., 2012). Briefly, genomic DNA was isolated from the buffy coat of EDTA treated whole blood samples (QIAGEN Inc., Valencia, CA). Diluted DNA (20 ng/μL) served as the template for polymerase chain reaction with specific primers [forward (5′-CAACAACATGAATCAGCCAGA-3′) and reverse (5′-CCTACATTCCTGTGTGTGCAA-3′)]. The 565-base pair amplicon was within the coding sequence of bovine *CYP3A28* [National Center for Biotechnology Information (NCBI) gi1769423; (Natsuhori et al., 1997)]. Purified amplification products (QIAGEN Inc., Valencia, CA) were restriction enzyme digested with *Alu* I (New England BioLabs, Beverly, MA). Steer genotypes at SNP site C994G were homozygous cytosine, homozygous guanine, and heterozygous.

#### Statistical analysis

Results of the CYP450 enzyme assay were reported in luminescence (arbitrary units). Pearson correlation coefficients were determined between dependent variables. Data were analyzed using mixed procedures with pasture as the experimental unit, genotype within pasture as random term, fescue cultivar as repeated, and creatinine as a covariate. If *F*-test for main or interactive effects were significant (*P* < 0.05) then means were separated using multiple *t*-tests.

## Results

**Figure 1:**
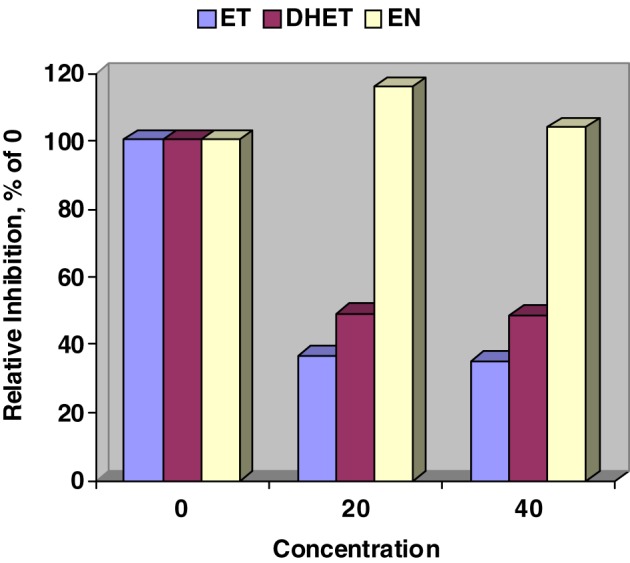
**Relative inhibition of *in vitro* cytochrome P450 activity by ergotamine (ET), dihydroergotamine (DHET), and ergonovine (EN)**. Interaction between alkaloid and concentration (μM) affected (*SE* = 4.67; *P* < 0.001) P450-Glo assay luminescence. Concentration of EN did not affect relative inhibition. Within ET and DHET 20 and 40 μM concentrations did not differ.

Figure 1 validates our previous work (Moubarak et al., 2012) that ET, and DHET inhibit CYP450 activity as detected by the Promega™ P450-Glo assay. In contrast, CYP450 activity was numerically stimulated when 20 and 40 μM of EN where added to the Glo assay. Our lowest test concentration, 20 μM, resulted in a steep inhibition of CYP450 by ET and DHET suggesting that the sensitivity of assay was exceeded.

Steer weight gain (0.53 ± 0.14 kg/d) was typical for summer grazing of tall fescue with light grain supplementation. Table 1 presents the Pearson correlation coefficients among body weight, gain, circulating concentrations of cortisol, prolactin, and IGF-1, urine inhibition of cytochrome P450 activity, and urine alkaloid concentrations. Cortisol concentrations at weaning were not correlated with body weight or gain. In contrast, prolactin concentrations and prolactin:cortisol were correlated (*r* > 0.30; *P* < 0.05) with body weight and ADG. Although steer body weights were correlated with IGF-1, ADG was not correlated to IGF-1. Inhibition of CYP450 activity by urine was correlated (*r* = −0.50; *P* < 0.05) with total ergot alkaloid concentration. Inhibition of CYP450 activity by urine also tended to be correlated (*r* = −0.26; *P* < 0.10) with ADG.

**Table 1:** **Correlations of steer weight and gain with serum hormones and urine alkaloids**.

**Item^a^**	**Mean ± SE**	**Weaning weight, kg 203 ± 27**	**End weight, kg 258 ± 34**	**ADG, kg 0.53 ± 0.14**
Cortisol, ng/mL	36.1±11.6	−0.05	−0.01	0.07
Prolactin, ng/mL	35.9±31.9	0.31^*^	0.46^**^	0.51^**^
Prl:cortisol	1.2±1.6	0.34^*^	0.41^**^	0.31^*^
IGF-1	197.1±46.6	0.48^**^	0.47^**^	0.20
Urine inhibition, %	3.7±2.7	0.03	−0.07	−0.26^+^
Urine alkaloids, ng/mg	331.1±325.7	0.04	−0.02	0.21

a *Steers (n = 39) were weaned and grazed mixed tall fescue pastures for 105 days. Serum concentrations of cortisol, prolactin, and insulin-like growth factor 1 were determined at weaning. Urine was collected at day 105 of summer grazing, effects of urine on in vitro CYP450 expressed as percent of control (Urine inhibition), and total ergot alkaloids in urine (Urine alkaloids) expressed as ng of alkaloids per mg of creatinine*.

+ *P < 0.1*.

* *P < 0.05*.

** *P < 0.01*.

**Figure 2:**
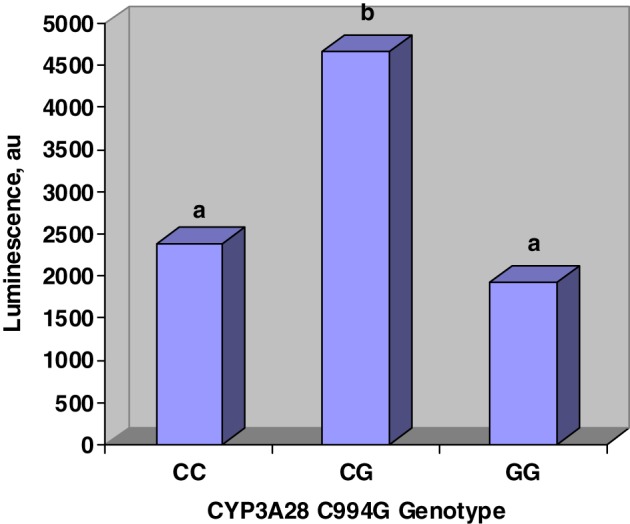
**Main effects of CYP3A28 genotype on CYP450 activity *in vitro***. Genotype was determined at C994G mutation. Urine was incubated in P450-Glo assay and luminescence was directly proportional to CYP450 activity. Genotype affected (*SE* = 770; *P* < 0.03) urine inhibition of CYP450 activity. Fescue cultivar did not affect (*P* > 0.1) luminescence.

Figure 2 displays the effects of C994G genotype on inhibition of CYP450 activity by urine. Steers that were heterozygous at C994G had more luminescence than steers that were either CC or GG. Luminescence was directly proportional to CYP450 activity; therefore, our work suggests that CG steers excreted urine that was less inhibitory to CYP450 activity. Tall fescue cultivar did not affect (*P* > 0.1) urine inhibition of CYP450.

## Discussion

Determining the bioavailability of ergot alkaloids in the biological fluids of animals grazing tall fescue (E+) pastures infested with wild-type *N. coenophialum* is difficult. Previously, ergot alkaloids have been determined in cattle serum via HPLC (Moubarak et al., 1996), and in steer urine and bile (Stuedemann et al., 1998). Our results demonstrate that the Promega™ P450-Glo assay offers another method of indirectly determining ergot alkaloid concentrations in the urine of steers. Our preliminary research using the P450-Glo assay for the detection of animal to animal variation in circulating alkaloid concentrations proved too inconsistent to be useful (data not presented); however, refinement of the methodology may lead to a valid assay. Linearity of inhibition was problematic when testing cattle serum and plasma.

Stuedemann et al. (1998) successfully quantified total ergot alkaloid concentration in steer urine and bile using a validated ELISA. They found that 96% of all ergot alkaloids consumed were excreted through the urine. We analyzed urine from steers in our study using the same ELISA methods and found a strong negative correlation with luminescence from the Promega™ P450-Glo assay, suggesting the P450-Glo assay could indirectly determine animal clearance of ergot alkaloids. The ELISA method has been used primarily when scientists are interested in determining the concentration of total ergot alkaloids, not particular alkaloids (Stuedemann et al., 1998; Hill et al., 2000; Ayers et al., 2009). However, the ELISA assay lacks selectivity as it does not differentiate between ergot alkaloids which have necessitated the use of chromatographic methods to detect specific ergot alkaloids (Schultz et al., 2006; De Lorme et al., 2007; Foote et al., 2012; Wang et al., 2013). Schnitzius et al. (2001), compared HPLC and ELISA detection of ergot alkaloids and found the methods were inconsistent in their identification of alkaloids, concluding that both had their advantages and disadvantages. Antigen (ergot alkaloids) detection is restricted to the specificity of the antibody used in the ELISA test.

The P450-Glo assay also lacks selectivity as any compound that will inhibit CYP450 enzymes will be detected. However, the antibody used for ELISA is specific for the lysergic acid moiety (Hill and Agee, 1994), meaning any precursor molecules present, such as clavines, will not be detected using ELISA, but they would be detected in the P450-Glo assay if they inhibit CYP450. Based on our research, we cannot predict which alkaloid(s) will be associated with animal toxicosis. In fact, it is interesting that one of the more water-soluble ergot alkaloids, ergonovine, did not inhibit the P450-Glo assay yet factors in the urine did inhibit formation of luminescence. Perhaps other water-soluble alkaloids such as clavines and(or) lysergic acid and its derivatives are accumulating in urine, that would be consistent with previous metabolic research (Ayers et al., 2009). Ergopeptine alkaloids (e.g., ergovaline) are not excreted in the urine, so they would not be there to inhibit CYP450 (Schultz et al., 2006; De Lorme et al., 2007; Merrill et al., 2007); however, their metabolites may be in the urine following phase 1 and phase 2 toxin clearance in the liver.

Selecting livestock that are less susceptible to ergot alkaloid poisoning has the potential to improve livestock production where tall fescue is a component of the grazing system. In reality that is what livestock producers have been doing for the last 60 years. If the primary forage is tall fescue for a producer and the livestock do not reproduce then they are culled which results in selection for animals that tolerate the ergot alkaloids. Previously, we demonstrated that mutations in bovine cytochrome *P4503A28* were associated with cattle profitability traits (Sales et al., 2012, 2013). In the current study, steer genotype at SNP C994G was associated with urine inhibition of CYP450 activity. Steers that were CG at C994G excreted urine that was less inhibitory to CYP450 activity. We speculate that heterozygous steers had metabolically altered the alkaloids which resulted in compounds that were not inhibitory of CYP450. Alternatively, CG steers may have deposited alkaloids in fat stores and were not excreted in the urine. Additional research will be required to determine ergot alkaloid deposition, and the dynamics and sensitivity of the P450-Glo-assay to biological fluids from cattle grazing various tall fescue cultivars.

The overall goal of our research is to develop management tools that can increase the sustainability of livestock production. In addition, we are interested in understanding the genetic and physiological mechanisms related to animal productivity and toxicology. Our results suggest that cytochrome P450 can be used as management tool for selecting animals based on their cytochrome *P4503A28* genotype. In addition, we can envision the use of the P450-Glo-assay as a selection assay. We have not tested our hypothesis, but it is plausible that a group of replacement animals could be purposely exposed to a large concentration of ergot alkaloids and urine monitored for inhibition of the P450-Glo-assay. Presumably those animals with less inhibitory urine would be those with more tolerance of ergot alkaloids, but that would need to be tested before being recommended as a selection practice.

### Conflict of interest statement

The authors declare that the research was conducted in the absence of any commercial or financial relationships that could be construed as a potential conflict of interest.
